# Cytochrome P450s-Involved Enhanced Metabolism Contributes to the High Level of Nicosulfuron Resistance in *Digitaria sanguinalis* from China

**DOI:** 10.3390/biology12091192

**Published:** 2023-08-31

**Authors:** Xumiao Wang, Wei Hu, Yuxi Li, Minghao Jiang, Ning Zhao, Haiqun Cao, Min Liao

**Affiliations:** 1 Anhui Province Key Laboratory of Crop Integrated Pest Management, School of Plant Protection, Anhui Agricultural University, Hefei 230036, China; wangxumiao@stu.ahau.edu.cn (X.W.); 22723183@stu.ahau.edu.cn (W.H.); liyuxi@stu.ahau.edu.cn (Y.L.); jiangmh@stu.ahau.edu.cn (M.J.); zhaon@ahau.edu.cn (N.Z.); caohaiqun@ahau.edu.cn (H.C.); 2 Anhui Province Engineering Laboratory for Green Pesticide Development and Application, School of Plant Protection, Anhui Agricultural University, Hefei 230036, China

**Keywords:** *Digitaria sanguinalis*, nicosulfuron, cross- and multiple resistance, P450s, metabolic resistance

## Abstract

**Simple Summary:**

*Digitaria sanguinalis* is considered one of the most malignant grass weeds worldwide and is widely distributed in Chinese maize fields. In this study, we explored the mechanism of metabolic resistance via cytochrome P450s of the LJ-01 population of *Digitaria sanguinalis*, which evolved high-level resistance to the ALS inhibitor nicosulfuron and evolved cross- and multiple resistance to thifensulfuron-methyl and pyrazosulfuron-ethyl, as well as the PS II inhibitors bromoxynil octanoate and atrazine. This study provides scientific and theoretical guidance for the control of resistant populations in *D. sanguinalis*.

**Abstract:**

Large crabgrass (*Digitaria sanguinalis* (L.) Scop.) is one of the major malignant grass weeds in Chinese maize (*Zea mays* L.) fields, and it has recently developed resistance to the acetolactate synthase (ALS)-inhibiting herbicide nicosulfuron. This study focused on a suspected nicosulfuron-resistant (R) population (LJ-01) of *D. sanguinalis*, collected from Lujiang County in Anhui Province, China, to explore the resistance level and potential resistance mechanism. Whole-plant dose–response testing confirmed that the LJ-01 population evolved a high level of resistance to nicosulfuron (11.5-fold) compared to the susceptible (S) population, DY-02. The *ALS* gene sequencing and relative expression assay of the R plants indicated that target gene mutation and overexpression were not responsible for the resistance phenotype. However, pretreatment with malathion, a known cytochrome P450 monooxygenase (P450) inhibitor, alleviated the resistance of the R population to nicosulfuron by approximately 36%. High-performance liquid chromatography (HPLC) analysis revealed that the R plants metabolized nicosulfuron faster than the S plants. Moreover, cross-resistance testing suggested that the R population exhibited low levels of resistance to thifensulfuron-methyl and pyrazosulfuron-ethyl, but it remained susceptible to rimsulfuron. Multiple resistance patterns showed that the R population evolved low resistance to the photosystem inhibitors bromoxynil octanoate and atrazine and sensitivity to the acetyl-CoA carboxylase (ACCase) inhibitor cyhalofop-butyl and the 4-hydroxyphenylpyruvate dioxygenase (HPPD) inhibitors tembotrione, mesotrione, and topramezone. This study reports, for the first time, the simultaneous resistance to ALS and different photosystem inhibitors in *D. sanguinalis*. The nicosulfuron resistance observed in the R population could primarily be attributed to an enhanced metabolism involving P450 enzymes.

## 1. Introduction

Maize (*Zea mays* L.) is one of China’s most important crops, and weeds affect maize yields through competition for relevant natural resources, such as light, water, and fertilizer [1,2]. Large crabgrass (*Digitaria sanguinalis* (L.) Scop.), a C4 plant with strong competitive ability and extensive ecological adaptability, has become one of the most noxious grass weeds in maize fields in China [3,4]. Acetolactate synthase (ALS)-inhibiting herbicides have been widely used to control annual grass and/or broadleaved weeds over the last two decades. Generally, ALS herbicides can be divided into five chemical classes, including sulfonylureas (SUs), imidazolinones (IMIs), triazolopyrimidines (TPs), pyrimidinothiobenzoates (PTBs), and sulfonylamino-carbonyl-triazolinones (SCTs) [5]. Among them, the SU herbicide nicosulfuron has been frequently used for POST control of grass weeds in maize fields, including *D. sanguinalis* [6]. Nevertheless, its excessive use has resulted in the problem of the “3Rs” (i.e., residue, resistance, and resurgence), and nicosulfuron resistance has caused major trouble in Chinese maize yields [6,7,8,9,10,11].

How weeds evolve resistance to herbicides can be divided into two major mechanisms: target-site resistance (TSR) and nontarget-site resistance (NTSR) [12]. For TSR, mutations in genes encoding the target proteins of a herbicide may be the main cause of ALS resistance, and it usually evolves cross-resistance to different herbicides with the same mode of action (MOA) [13]. To date, a total of nine codon positions in *ALS* have been reported [14,15]. Target-site mutations could confer a high level of resistance in *D. sanguinalis* [16]. Target gene overexpression is another TSR mechanism of ALS inhibitors. For example, the high level of resistance of different ALS inhibitors may be related to the overexpression of the *ALS* gene in the resistant population of barren brome (*Bromus sterilis* L.) compared to the susceptible population [17]. In comparison, NTSR is characterized by changes in absorption, translocation, excretion, or sequestration, as well as an increased metabolic degradation, and it can often endow resistance to multiple herbicides within the same, or even different, groups of herbicides [18,19]. Of these, an enhanced herbicide metabolism (i.e., metabolic resistance) plays a major role in NTSR evolution [20]. Metabolic resistance often involves several common detoxification enzymes, including cytochrome P450 monooxygenases (P450s), glutathione S-transferases (GSTs), UDP-glycosyltransferases (UDP-GTs), and ATP-binding cassette (ABC) transporters. A previous study reported that enhanced P450 enzyme activity, thus an increased herbicide metabolism, caused ALS resistance in *D. sanguinalis* [3,21].

In this study, a *D. sanguinalis* population, namely, LJ-01, was collected from a maize field in Anhui Province that had failed at controlling nicosulfuron at its field recommended rate (FRR) of 60 g a.i. ha^−1^. Based on the suspected herbicide-resistant population, the aims of this study were to (1) investigate its susceptibility and resistance level to nicosulfuron; (2) explore its internal molecular mechanisms responsible for the nicosulfuron resistance; and (3) characterize its cross- and multiple resistance pattern to other herbicides.

## 2. Materials and Methods

### 2.1. Plant Materials and Growth Conditions

Two *D. sanguinalis* populations were used in this study, including a putative nicosulfuron-resistant (R) population, LJ-01, and a susceptible (S) population, DY-02. The R population was randomly harvested from a maize field in Lujiang County, Anhui Province (31.48° N, 117.22° E), in October 2021, with a known history of controlling *D. sanguinalis* with ALS inhibitors. The S population was gathered from fallow land in Dayang County, Anhui Province (31.93° N, 117.21° E), in August 2022, with no history of herbicide application. Mature seeds from each population were obtained from ≥200 individual plants. These seeds were air-dried and stored in paper bags at 4 °C until use.

Seeds from different large crabgrass plants of the R and S populations were placed in two layers of filter paper humidified with deionized water and incubated in a growth incubator with a constant temperature of 30 °C with a 12 h photoperiod. Following the seeds’ germination after 4 days, 12 seedlings were transplanted into individual, separate square plastic pots (9 cm × 9 cm × 7 cm), which were then filled with loam soil and nurtured in a greenhouse under controlled conditions for natural light, with the temperatures maintained at 35 °C during the day and 25 °C during the night, with approximately 75% relative humidity. In order to provide an adequate level of soil moisture, the plastic pots were watered on alternate days. The number of seedlings per pot was reduced to six plants when they reached the two- to three-leaf stage.

### 2.2. Herbicides and Chemicals

A total of 10 herbicides with four distinct modes of action (MOAs) were used in whole-plant dose–response experiments. Their information is shown in Table 1. Malathion (99%), purchased from Sigma-Aldrich (Shanghai, China), was used as a P450 inhibitor.

### 2.3. Whole-Plant Dose–Response Experiments

Whole-plant dose–response experiments were carried out to determine the susceptibility and resistance level to nicosulfuron in the R and S populations. Nicosulfuron was sprayed on the R and S seedlings at various series of doses when they were in the three- to four-leaf stages (Table 1). The application of all herbicides was carried out 6 days after the weeds were transplanted using a laboratory cabinet sprayer (3WP-2000, Nanjing Mechanization Research Institute of the Ministry of Agriculture, Nanjing, China) delivering 450 L ha^−1^ at 0.275 MPa. After growing for another 21 d under the same conditions, the fresh weights of the aboveground shoots for each pot were measured and recorded.

### 2.4. Identification of Mutations in the ALS Gene

To identify *ALS* gene mutations in the R population of *D. sanguinalis*, a total of 10 plants were randomly selected from the R and S populations and, subsequently, cultivated until reaching the three- to four-leaf stages. Genomics DNA were then isolated from the fresh leaf tissues harvested from each individual plant using the cetyltrimethylammonium bromide (CTAB) method [22]. Four primer pairs, containing all nine *ALS* gene mutations sites, as described by Mei et al. [3], were used to amplify the *ALS* genes from both the S and R plants using polymerase chain reaction (PCR) (Table S1). These DNA fragments were then assembled into a 1863 bp sequence. PCR amplification was used with the 2 × Super Pfx MasterMix (CWBIO, Beijing, China) and the programs were as follows: 3 min at 98 °C, 35 cycles of 30 s at 98 °C, 30 s at 60 °C (depending on the primer pairs), 30 s at 72 °C, and 10 min at 72 °C for the final extension. Subsequently, the PCR products were seen using 1% agarose gel and afterwards subjected to sequencing analysis conducted by Tsingke Biotech (Beijing, China). The obtained *ALS* gene sequence data were aligned and analyzed in comparison to the full-length *ALS* sequence of *Arabidopsis thaliana* using DNAMAN v.6.0 software (Lynnon Biosoft, Vaudreuil city, QC, Canada).

### 2.5. Analysis of the Expression Level of the ALS Gene

The R and S seedlings of *D. sanguinalis* at the three- to four-leaf stages were sprayed with nicosulfuron at an FRR of 60 g a.i. ha^−1^, and their fresh leaves were collected at 0, 12, and 24 h after treatment (HAT). A TRIzol reagent kit (Invitro-180 gen, Carlsbad, CA, USA) was used to isolate the total RNA of each leaf sample. The cDNA was synthesized using TransScript One-Step gDNA Removal and cDNA Synthesis SuperMix (TransGen Biotech, Beijing, China). Real-time quantitative PCR (rt-qPCR) was performed using a ChamQ SYBR qPCR Master Mix Kit (Vazyme, Nanjing, China), and the primers for the qPCR were designed using Primer Premier v.5.0 software (PREMIER Biosoft International, Palo Alto, San Francisco city, CA, USA) (Table S1). The amplification protocol consisted of 30 s at 95 °C followed by 40 cycles of 10 s at 95 °C and 30 s at 60 °C. The *ALS* expression relative to that of the reference gene (qActin-F/R) was analyzed using the 2^−∆∆Ct^ method [23]. The experimental design consisted of three biological duplicates (with each replication consisting of samples obtained from an individual plant), and three technical duplicates were performed for each biological replicate. Two threshold values, the Student’s *t*-test (*p* < 0.05) and a 2-fold change, were adopted to analyze the up- or downregulation of the *ALS* expression.

### 2.6. Susceptibility Level to Nicosulfuron after P450 Inhibitor Treatment

These tests were carried out at the same time as the whole-plant dose–response experiments. At the three- to four-leaf stages, malathion and malathion plus nicosulfuron were applied to the R and S seedlings, respectively. Malathion at a dose of 1000 g a.i. ha^−1^ was sprayed 1 h before nicosulfuron was applied, while nicosulfuron was applied in the same series of doses, as shown in Table 1. All herbicides and chemicals were sprayed as described in Section 2.3. The aboveground tissues were harvested at 21 d after treatment (DAT), and the fresh weight of the samples was measured and recorded.

### 2.7. Detection of Nicosulfuron Residues in R and S Plants

To compare the variety of the metabolic rates of nicosulfuron, the S and R plants were nurtured to the three- to four-leaf stages under the abovementioned conditions and were foliar treated with nicosulfuron at an FRR of 60 g a.i. ha^−1^. Technical material nicosulfuron (95%), purchased from Yuanye Biotechnology Co., Ltd. (Shanghai, China), was used for the testing, and it was dissolved using HPLC-grade methanol and diluted with 0.1% Tween-80 aqueous emulsions to the dose of the FRR. The control plants were subjected to the application of aqueous emulsions containing equal amounts of methanol and Tween-80. The application of all solutions was via spraying using a laboratory cabinet sprayer, according to Section 2.3. The aboveground tissues for the S and R plants were obtained at 6, 12, 24 and 48 HAT, rapidly frozen in liquid nitrogen, and stored at −80 °C. The experiment contained three biological replicates, each with six plants from the same pot.

The extraction of nicosulfuron from each sample was performed using the procedures reported by Yu et al. [3]. The sample separation and detection were conducted using an LC-20AT liquid chromatography (HPLC) system (Agilent Corp., Santa Clara, San Francisco city, CA, USA) equipped with a C_18_ column (4.6 mm × 250 mm, 5 μm; Agilent Corp.). The mobile phase was composed of 70% acetonitrile and 30% ultrapure water (*v*/*v*) with 0.2% formic acid. The injection volume was 10 μL, the flow rate was set at 1 mL min^−1^, the column temperature was held at 30 °C, and the compound was detected at 254 nm.

Linearity was established in accordance with the criteria set out by nicosulfuron at concentrations of 0.10, 0.20, 0.50, 0.80, 1.00, 1.50 and 2.00 mg L^−1^. The determination of the relative standard deviations (RSDs) was conducted based on the recovery tests using standard spiked samples (n = 5) at levels of 1.00, 2.00, and 5.00 µg g^−1^ and used to assess the precision.

### 2.8. Resistance Pattern to Different Herbicides

Whole-plant dose–response experiments were conducted to determine the resistance levels in the R population to herbicides with same and different MOAs. Three ALS inhibitors (thifensulfuron-methyl, rimsulfuron, and pyrazosulfuron-ethyl), three HPPD inhibitors (tembotrione, mesotrione, and topramezone), two photosystem II (PS II) inhibitors (atrazine and bromoxynil), and an ACCase inhibitor (cyhalofop-butyl) were used for the testing, and the dosages were identified based on preliminary tests, which are presented in Table 1. The herbicides’ application was the same as described in Section 2.3. The aboveground fresh weights per pot were recorded at 21 DAT.

### 2.9. Data Analyses

All whole-plant dose–response experiments were performed twice, with each treatment containing three biological replicates. The ANOVA data showed no differences (*p* > 0.05) between the repeated experiments; thus, the results of the same trials were pooled, and a nonlinear regression analysis was carried out on the data. The herbicide dose inhibition of plant growth by 50% (GR_50_) was computed using the following four-parameter nonlinear logistic curve (Equation (1)) in the SigmaPlot v.14.0 software (Systat Software, San Jose, CA, USA).

(1) $$y=C+\frac{D-C}{1+(\frac{x}{GR_{50}}{)}^{b}}$$

In this equation, y represents the response to each herbicide at dose x, b is the slope around GR_50_, and *C* and *D* are the lower and upper limits of the response, respectively. The resistance index (RI), an estimation of the level of herbicide resistance, was determined by comparing the GR_50_ value of the resistance population to that of the susceptible population.

## 3. Results

### 3.1. Susceptibility to Nicosulfuron in R and S Plants

The GR_50_ value of the S population, DY-02, was 6.7 g a.i. ha^−1^, which was much lower than its FRR of 60 g a.i. ha^−1^ (Table 2). However, the GR_50_ value of the R population, LJ-01, to nicosulfuron was 76.8 g a.i. ha^−1^, which was 11.5-fold that of the S population (Table 2).

### 3.2. Sequencing and Analysis of ALS Gene

**Figure 1:**
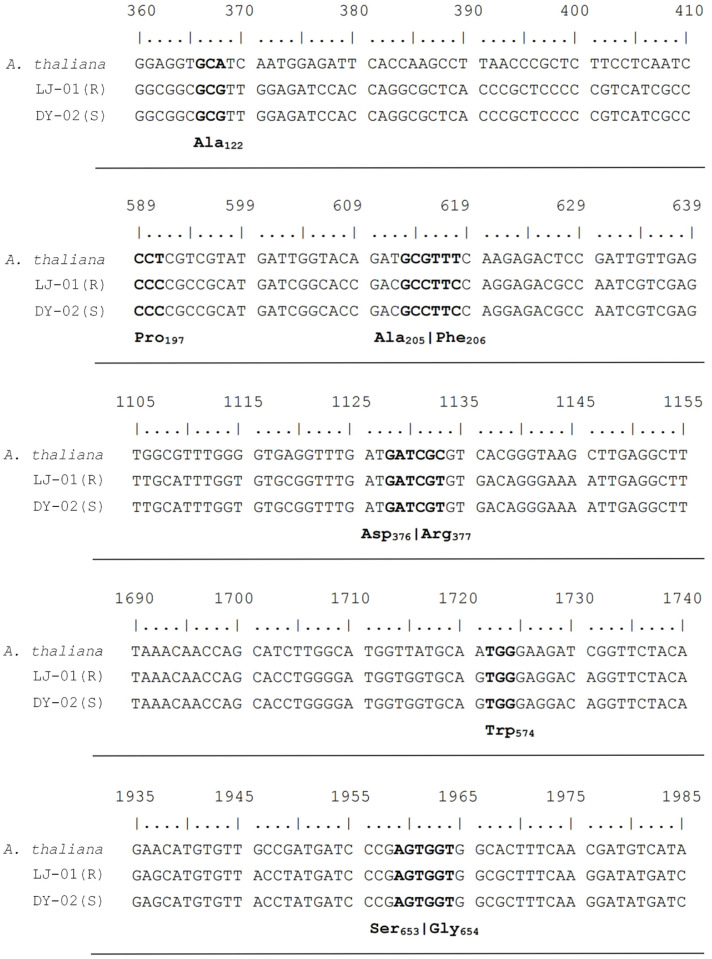
Sequence alignment of *ALS* genes amplified from the ALS-resistant (LJ-01) and ALS-susceptible (DY-02) populations of *D. sanguinalis*. The bold text indicates the known nine codon positions in *ALS* that can mutate to confer ALS resistance.

The results of the sequencing and comparison of the *ALS* gene did not show differences between the R and S *D. sanguinalis* plants in the codons Ala122, Pro197, Ala205, Phe206, Asp376, Arg377, Trp574, Ser653, and Gly654 (Figure 1).

**Figure 2:**
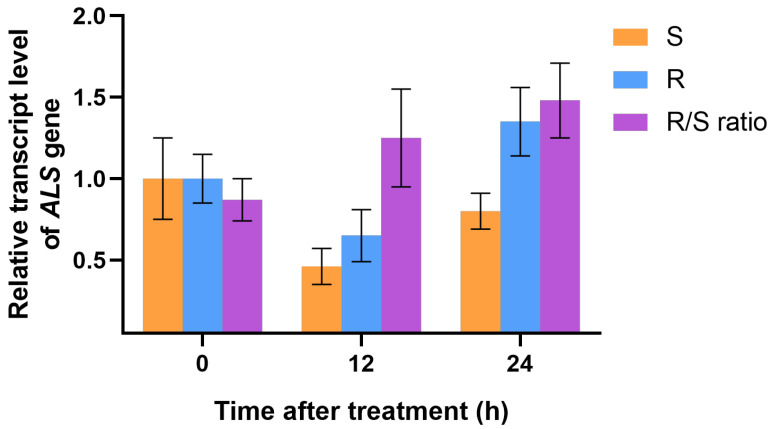
Relative expression level of the *ALS* gene in the R and S plants of *D. sanguinalis* at 0 (control), 12, and 24 h after nicosulfuron treatment. No significant difference in *ALS* expression between R and S populations was detected. Vertical bars represent the standard errors of the means.

There were no differences in the *ALS* gene expression (fold change < 2; *p >* 0.05) between untreated R and S plants and at 12 and 24 HAT after nicosulfuron treatment (Figure 2). This result suggests that *ALS* overexpression is not involved in nicosulfuron resistance in R.

### 3.3. Effects of Malathion on Nicosulfuron Resistance

**Figure 3:**
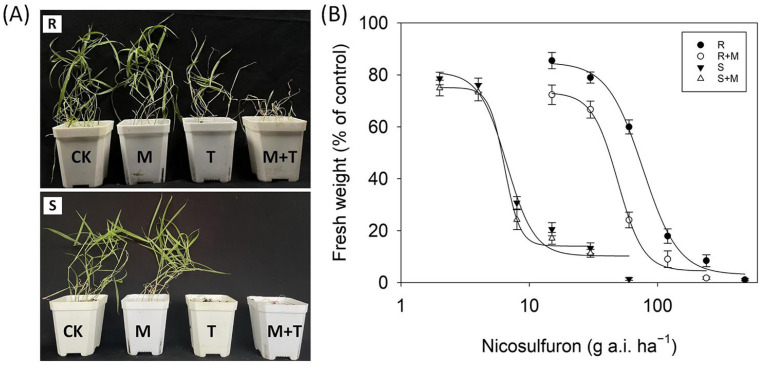
(**A**) Nicosulfuron susceptibility of the S (bottom) and R (top) populations without or with P450 inhibition. Weed seedlings were grown to the three- to four-leaf stages and treated with water (CK), malathion alone (M), nicosulfuron alone (T), and malathion plus nicosulfuron (M + T). Photographs were taken 21 d after treatment. (**B**) Whole-plant dose–response curves for the aboveground fresh weights of the S (▼) and R (●) populations treated with nicosulfuron alone or nicosulfuron plus 1000 g a.i. ha^−1^ malathion (△, ○). Vertical bars represent the standard errors of the means.

The growth of the R and S plants treated with the P450 inhibitor malathion alone was not affected (Figure 3). For the S population, the GR_50_ values to nicosulfuron were 6.7 and 6.4 g a.i. ha^−1^ (Table 2) with or without malathion pretreatment, respectively, indicating that inhibiting P450 activity does not affect its susceptibility. In contrast, for the R population, malathion pretreatment reduced its resistance to nicosulfuron by approximately 36%, with the GR_50_ values reduced from 76.8 to 49.1 g a.i. ha^−1^ (Table 2). This suggests that P450s were most likely involved in the nicosulfuron-resistance phenotype in the R plants.

### 3.4. Nicosulfuron Metabolism in D. sanguinalis

**Figure 4:**
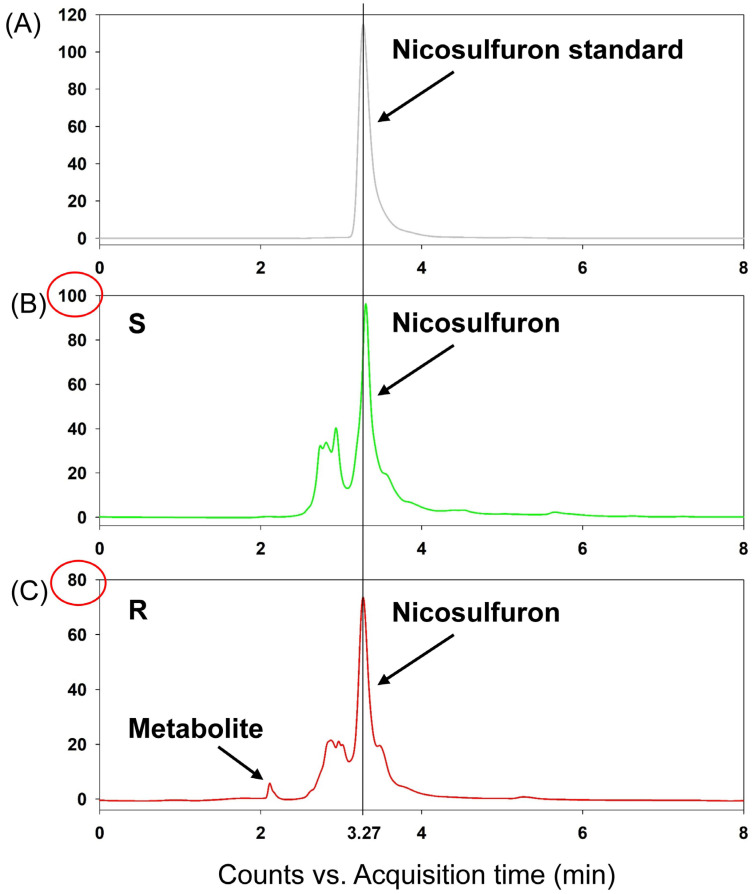
Typical chromatograms of the nicosulfuron detected in the (**A**) standard, (**B**) green means S plants, and (**C**) red means R plants at 48 h after treatment.

In the current study, nicosulfuron was well separated and detected using HPLC, and its retention time was 3.27 min (Figure 4). The recovery (76% to 99%) and RSD (2.49% to 3.51%) values suggest that this analysis method was credible for quantification.

The absorption of nicosulfuron was similar in both the R and S plants, because the amount recorded in the S plants displayed no differences with the R plants until 12 HAT (Table 3) [24]. However, the analysis revealed higher levels of nicosulfuron in the S plants compared to the R plants at both 24 and 48 HAT. Notably, when comparing the chromatogram of the S plants at 48 HAT, one more peak with a retention time of 3.27 min appeared in the chromatogram of the R plants (Figure 4).

### 3.5. Cross- and Multiple Resistance to Other Herbicides

**Figure 5:**
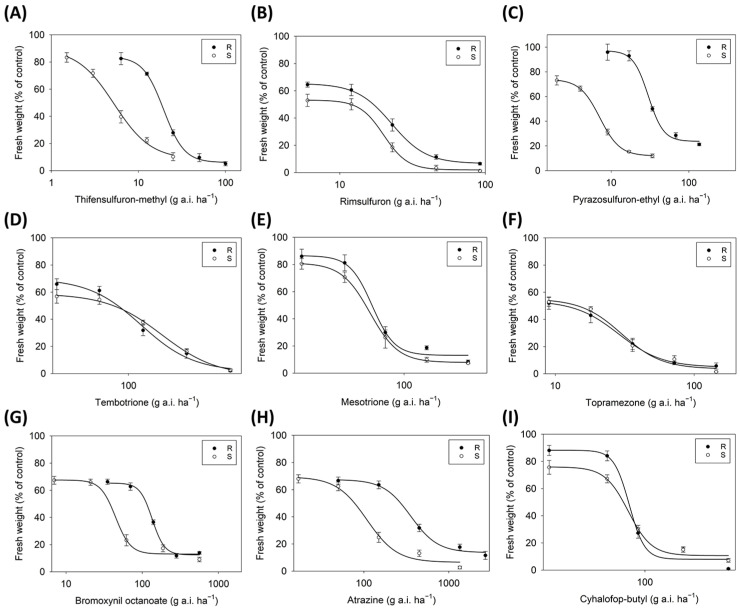
Susceptibilities of the R and S populations of *D. sanguinalis* to nine herbicides with four modes of action, including ALS inhibitors: (**A**) thifensulfuron-methyl; (**B**) rimsulfuron; (**C**) pyrazosulfuron-ethyl; HPPD inhibitors—(**D**) tembotrione, (**E**) mesotrione, and (**F**) topramezone; PS II inhibitors—(**G**) bromoxynil octanoate and (**H**) atrazine; ACCase inhibitor—(**I**) cyhalofop-butyl. Vertical bars represent the standard errors of the means.

The whole-plant dose–response experiments indicated that the R population, LJ-01, also evolved resistance to some of the herbicides tested, whereas the S population, DY-02, was susceptible to all of the herbicides tested. Based on the RI values, LJ-01 was 3.7-fold more resistant to thifensulfuron-methyl (Figure 5A), 4.2-fold more resistant to pyrazosulfuron-ethyl (Figure 5C), 3.0-fold more resistant to bromoxynil octanoate (Figure 5G), and 3.3-fold more resistant to atrazine (Figure 5H) than DY-02 (Table 4). However, LJ-01 remained susceptible to rimsulfuron (Figure 5B), tembotrione (Figure 5D), mesotrione (Figure 5E), topramezone (Figure 5F), and cyhalofop-butyl (Figure 5I), with RIs ranging from 0.7 to 1.1 (Table 4).

## 4. Discussion

However, resistance to nicosulfuron has been reported in *D. sanguinalis* in recent years due to its excessive use [3,16,25]. It has been observed that farmland weeds can rapidly evolve resistance to ALS inhibitors with continuous and excessive use for several years [26,27]. In this study, the *D. sanguinalis* population LJ-02, which survived nicosulfuron at the FRR, was harvested from a maize field in Anhui Province. The whole-plant dose–response experiment indicated that the R plants conferred a high level of resistance to nicosulfuron (RI = 11.5). This indicates that LJ-01 evolved high resistance to nicosulfuron, while DY-02 was susceptible to it. According to local farmers, the field from which the R population was collected has been continually treated with ALS herbicides for more than 10 years. This suggests that the long-term use of a single herbicide inevitably facilitates resistance evolution [14].

TSR is an important reason for the resistance of weeds to herbicides with the same MOA [28]. Target-site mutations in the *ALS* gene that cause many farmland weed species to survive the application of ALS herbicides have been reported [29,30,31]. In recent studies, a mutation was found to confer resistance to ALS-inhibiting herbicides in *D. sanguinalis*. [25]. In this study, no known *ALS* mutations were found in the R plants, indicating that *ALS* mutation is not the internal resistance mechanism in the R plants (Figure 1). The reduced affinity of ALS herbicides to target sites can also be caused by target gene overexpression, leading to an increased number of enzymes being produced after herbicide treatment in order to avoid herbicide damage [32]. Sen et al. [17] reported that the ALS resistance in a population of *B. sterilis* was contributed to both by enhanced P450 activity and *ALS* overexpression. Zhao et al. [33] reported a population of mesosulfuron-methyl-resistant shortawn foxtail (*Alopecurus aequalis* Sobol.), which was endowed by gene overexpression, and the qRT-PCR revealed that the expression in the *ALS* gene was 21.0- and 15.5-fold upregulated in the ALS-resistant population. However, in this study, no difference (fold change < 2; *p* > 0.05) was observed in the relative expression level of the *ALS* gene between the R and S biotypes at 0 (untreated), 12, and 24 HAT (Figure 2) with or without nicosulfuron treatment, indicating that *ALS* overexpression is not involved in the nicosulfuron resistance in the R plants. These results indicate that nicosulfuron resistance in *D. sanguinalis* is not TSR.

NTSR is an important mechanism that could confer resistance to herbicides with different MOAs [34,35]. Since the late 1990s, P450s have been the key focus of research on resistance mechanisms [36]. The enhanced activity of common detoxification enzymes contributing to herbicide resistance has been identified in different weed species [37,38]. Malathion and NBD-Cl, the known P450 and GST inhibitors, respectively, are often used to investigate P450- and GST-mediated metabolic resistance in weed plants [39,40]. In this study, malathion pretreatment greatly reduced the level of resistance to nicosulfuron in the R population by approximately 36% (Figure 3), indicating that increased P450 activity is contributing to the resistance to the nicosulfuron phenotype in the R plants. Moreover, there was a difference in the amounts of nicosulfuron between the R and S plants of *D. sanguinalis* at 24 and 48 HAT. The R plants exhibited a higher rate of nicosulfuron metabolism compared to the S plants, and the enhanced nicosulfuron metabolism could be partly responsible for the resistance observed in the R plants. This indicates that the enhanced rates of nicosulfuron metabolism in the R plants may lead to the more rapid production of its potential metabolite. Similarly, Zhao et al. [41] reported that in the resistance population of Chinese sprangletop (*Leptochloa chinensis* (L.) Nees), the amount of cyhalofop acid was lower than in the susceptible plants, suggesting that an enhanced herbicide metabolism was involved in the resistance. Additionally, one more metabolite was detected in the R plants than in the S plants at 48 HAT, indicating that the enhanced P450 activity may have rapidly catalyzed the metabolism of nicosulfuron to other metabolites. However, the phytotoxicity of the metabolite remained unclear. Future studies are required to determine the structure of the potential metabolite and, thus, speculate on the crucial catalytic function of the P450s involved.

NTSR can frequently endow different MOA herbicides with unpredictable resistance, even for herbicides that have not yet been marketed [42]. In the current study, we also determined the resistance patterns of the R population to different herbicides and found that they exhibited low levels of cross-resistance to ALS inhibitors and multiple resistance to PSII inhibitors. Similarly, metabolic resistance was identified in diverse weed species, endowing them with uncertain patterns of multiple resistance, such as plumeless thistle (*Carduus acanthoides* L.) [43], Italian ryegrass (*Lolium multiflorum* L.) [44], and late watergrass *Echinochloa phyllopogon* (Stapf) Koss [45]. Although the R population, LJ-01, was also resistant to thifensulfuron-methyl, pyrazosulfuron-ethyl, bromoxynil octanoate, and atrazine, it remained susceptible to rimsulfuron, tembotrione, mesotrione, and topramezone. These susceptible herbicides could be used as alternatives to control the R population of *D. sanguinalis* in maize fields. However, it is worth noting that cyhalofop-butyl, which is highly efficient at controlling the R population, has not been registered for weed control in maize fields.

## 5. Conclusions

In summary, this study identified a metabolic–herbicide-resistant population of *D. sanguinalis*, LJ-01, that has evolved multiple resistance to different ALS and PSII herbicides. The nicosulfuron resistance in the resistant population was not conferred by target gene mutation and overexpression but could be mainly endowed by P450-mediated enhanced herbicide metabolism. Herbicide-resistant weeds are a great threat to crop production, which has mainly been attributed to the long-term use of single MOA herbicides. It is very important to apply herbicides with different MOAs and to develop an integrated weed management strategy to effectively control the resistant weeds.

## Figures and Tables

**Table 1:** Herbicides rates used in this study.

MOAs	Herbicides	Formulation	Supplier	Rate of Applications (g a.i. ha^−1^) ^a^	
				Susceptible Population	Resistant Population
ALS	Nicosulfuron	40 g L^−1^ OD	Vicome Greenland, Shandong	0, 2, 4, 8, 15, 30, **60**	0, 15, 30, **60**, 120, 240, 480
	Thifensulfuron-methyl	75% WDG	Ruibang, Jiangsu	0, 1.5, 3, 6.30, 12.5, **25**	0, 6.30, 12.5, **25**, 50, 100
	Rimsulfuron	25% WDG	Ruibang, Jiangsu	0, 6, 12, **23**, 46, 92	0, 6, 12, **23**, 46, 92
	Pyrazosulfuron-ethyl	15% OD	Shareworld, Anhui	0, 2, 4, 9, 17, **34**	0, 9, 17, **34**, 68, 136
HPPD	Tembotrione	8% OD	Jiuyi Agriculture, Anhui	0, 32, 63, **126**, 252, 504	0, 32, 63, **126**, 252, 504
	Mesotrione	15% SC	Xingyu Chemical, Hefei	0, 18, 37, 73, **146**, 292	0, 18, 37, 73, **146**, 292
	Topramezone	4% OD	Suncas, Hunan	0, 9, 18, **36**, 72, 144	0, 9, 18, **36**, 72, 144
PS II	Bromoxynil octanoate	25% EC	Ruibang, Jiangsu	0, 7, 21, 62, 187, **562**	0, 35, 70, 140, 281, **562**
	Atrazine	90% WDG	Tianyi Herbicide, Liaoning	0, 17, 50, 150,450, **1349**	0, 17, 50, 150,450, **1349**
ACCase	Cyhalofop-butyl	100 g L^−1^ EC	Yonon, Zhejiang	0, 23, 45, **90**, 180, 360	0, 23, 45, **90**, 180, 360

OD, oil dispersion; WDG, water dispersible granule; SC, suspension concentrate; EC, emulsifiable concentrate. ^a^ The field recommended rate for each herbicide is shown in bold.

**Table 2:** GR_50_ value used for the four-parameter log-logistic equation (Equation (1)) ^a^ and RI of large crabgrass populations in the R and S populations after nicosulfuron treatment.

Herbicide	Biotype ^b^	Regression Parameters				GR_50_ (g a.i. ha^−1^) (SEM)	RI ^c^
		*C* (SEM)	*D* (SEM)	b (SEM)	*R* ^2^		
Nicosulfuron	R	3.0 (2.8)	84.8 (3.0)	3.1 (0.5)	0.99	76.8 (5.0)	11.5
	S	10.2 (5.9)	81.2(10.3)	4.0 (2.6)	0.96	6.7 (1.3)	
Nicosulfuron plus malathion	R	4.5 (3.4)	73.2 (4.9)	4.4 (1.4)	0.99	49.1 (4.7)	7.7 *
	S	14.1 (2.9)	75.1 (4.0)	7.2 (4.2)	0.99	6.4 (0.9)	

^a^ Equation 1: y = C + (D − C)/(1 + (x/GR_50_) ^b^), where C and D are the lower and upper limits, respectively, and b is the slope around GR_50_. ^b^ R, resistant population LJ-01; S, susceptible population DY-02. ^c^ RI, resistant index. RI was calculated using the GR_50_ values of the resistant versus susceptible population. SEM, standard error of the means. * Significant difference (*p* < 0.05).

**Table 3:** Amounts of nicosulfuron detected in the R (LJ-01) and S (DY-02) plants at 6, 12, 24, and 48 HAT.

Time after Nicosulfuron Treatment (h)	Residual Amounts of Nicosulfuron (µg g^−1^)	
	R	S
6	5.74 ± 0.17 a	5.60 ± 0.19 a
12	5.03 ± 0.07 a	5.15 ± 0.13 a
24	3.58 ± 0.07 b	3.85 ± 0.10 a
48	2.56 ± 0.05 b	3.14 ± 0.12 a

Different letters with the same line for three repetitions are significantly different (*p* < 0.05). Each value represents the mean ± standard error.

**Table 4:** Parameter values of the four-parameter log-logistic equation (Equation (1)) ^a^ used to fit the plant growth response resulting from the nine herbicides tested.

Herbicide	Biotype ^b^	Regression Parameters				GR_50_ (g a.i. ha^−1^)(SEM)	RI ^c^
		*C* (SEM)	*D* (SEM)	b (SEM)	*R* ^2^		
Thifensulfuron-methyl	R	6.0 (1.4)	84.0 (2.2)	3.7 (0.4)	0.99	19.5 (0.7)	3.7
	S	8.3 (6.0)	89.8 (8.2)	2.1 (0.6)	0.99	5.3 (0.7)	
Rimsulfuron	R	6.6 (1.1)	65.4 (1.2)	3.6 (0.4)	0.99	22.7 (0.6)	1.1
	S	1.9 (0.8)	53.2 (1.1)	5.3 (0.7)	0.99	20.0 (0.6)	
Pyrazosulfuron-ethyl	R	23.7 (3.4)	97.2 (4.6)	4.5 (1.7)	0.99	30.1 (2.4)	4.2
	S	11.9 (0.4)	74.0 (0.5)	3.4 (0.1)	0.99	7.1 (0.1)	
Tembotrione	R	1.5 (8.1)	70.0 (8.8)	2.4 (1.2)	0.99	122.2 (22.4)	0.7
	S	−1.8 (3.8)	58.9 (2.2)	2.3 (0.4)	0.99	170.0 (13.2)	
Mesotrione	R	13.1 (5.1)	86.5 (7.2)	5.3 (2.7)	0.99	58.5 (8.3)	1.0
	S	7.9 (0.7)	81.2 (1.0)	4.2 (0.2)	0.99	56.5 (1.0)	
Topramezone	R	4.8 (0.7)	53.8 (1.1)	2.8 (0.2)	0.99	28.6 (0.8)	1.0
	S	3.5 (5.2)	55.2 (7.3)	3.0 (1.6)	0.98	30.0 (5.6)	
Bromoxynil octanoate	R	12.4 (2.3)	65.3 (2.6)	5.4 (3.2)	0.99	134.9 (6.6)	3.0
	S	13.1 (4.1)	67.6 (5.8)	4.4 (4.1)	0.98	44.6 (15.4)	
Atrazine	R	13.7 (3.0)	67.5 (4.0)	2.7 (1.1)	0.99	356.2 (54.0)	3.3
	S	6.5 (5.3)	69.5 (7.7)	2.5 (1.3)	0.98	108.3 (27.8)	
Cyhalofop-butyl	R	8.0 (7.1)	88.2 (10.0)	8.5 (7.1)	0.98	78.6 (11.0)	1.0
	S	10.7 (3.8)	76.0 (5.3)	5.6 (1.9)	0.99	77.4 (6.2)	

^a^ Equation 1: y = *C* + (*D* − *C*)/(1 + (x/GR_50_) ^b^), where *C* and *D* are the lower and upper limits, respectively, and b is the slope around GR_50_. ^b^ R, resistant population LJ-01; S, susceptible population DY-02. ^c^ RI, resistant index. RI was calculated using the GR_50_ values of the resistant versus susceptible population. SEM, standard error of the means.

## Data Availability

The data presented in this study are available upon request from the corresponding author.

## Supplementary Materials

The following supporting information can be downloaded at: https://www.mdpi.com/article/10.3390/biology12091192/s1, Table S1: Primer sequences of the *ALS* gene to measured mutation and expression levels in susceptible and resistant populations.
