# Supplementary material for: Cytochrome P450s-Involved Enhanced Metabolism Contributes to the High Level of Nicosulfuron Resistance in Digitaria sanguinalis from China
Source: Biology (Basel). 2023 Aug 31;12(9):1192. doi: 10.3390/biology12091192 (PMC10525941; doi:10.3390/biology12091192)
Supplement: Supplementary file 1 [file biology-12-01192-s001.zip › biology-2548665-supplementary.pdf]

Table S1. Primer sequences of *ALS* gene to measured mutation and expression levels in susceptible and resistant populations.

Primers	Sequence (5’ -3’)	Product Size (bp)	Annealing Temperature (°C)	Usage
ALS-F1	CGACGTCTTCGCCTACCC	451	60	Sequencing for *ALS*
ALS-R1	AGCCATCTGCTGTTGGATGT			
ALS-F2	GTCATCGCCAACCACCTCT	495		
ALS-R2	CGACTCACCAACAAGACGC			
ALS-F3	CCCCAAGGACATCCAGCAG	780		
ALS-R3	CCCATAGCCCCAAGACCAG			
ALS-F4	GTTGGGCAGCACCAGATGT	750		
ALS-R4	AAGCTACTTAAGATTACCATACCAGAGT			
qALS-F	GTGACGACCCACTGTCTCTC	110		*ALS* expression
qALS-R	ATCATCAAACCGCACACC			
qActin-F	AATCGGTGCAGAGAGGTTC	93		Reference
qActin-R	ATGATGGAGTTGTATGTGGC			
